# Cytogenetic characterization of four species of the genus *Hypostomus* Lacépède, 1803 (Siluriformes, Loricariidae) with comments on its chromosomal diversity

**DOI:** 10.3897/CompCytogen.v5i5.1589

**Published:** 2011-12-22

**Authors:** Marceléia Rubert, Renata da Rosa, Fernando Camargo Jerep, Luiz Antônio Carlos Bertollo, Lucia Giuliano-Caetano

**Affiliations:** 1 Departamento de Genética e Evolução, Universidade Federal de São Carlos, Rodovia Washington Luís, km 235 - SP-310, P.O. Box 676, CEP 13565-905, São Carlos, São Paulo, Brazil; 2 Departamento de Biologia Geral, Universidade Estadual de Londrina, Rodovia Celso Garcia Cid, Pr 445 Km 380, Campus Universitário, P.O. Box 6001, CEP 86051-970, Londrina, Paraná, Brazil; 3 Museu de Ciências e Tecnologia, Pontifícia Universidade Católica, Av. Ipiranga, 6681, P.O. Box 1429, CEP 90619-900, Porto Alegre, Rio Grande do Sul, Brazil

**Keywords:** loricariid catfishes, chromosome banding, NORs, fluorochromes, fluorescence in situ hybridization (FISH)

## Abstract

Cytogenetic analyses were performed on fishes of the genus *Hypostomus* (*Hypostomus ancistroides* (Ihering, 1911), *Hypostomus strigaticeps* (Regan, 1908), *Hypostomus regani* (Ihering, 1905), and *Hypostomus paulinus* (Ihering, 1905)) from the seven tributaries of the Paranapanema River Basin (Brazil) by means of different staining techniques (C-, Ag-, CMA_3_- and DAPI-banding) and fluorescence in situ hybridization (FISH) to detect 18S rDNA sites. All species showed different diploid numbers: 2n=68 (10m+26sm+32st-a) in *Hypostomus ancistroides*, 2n=72 (10m+16sm+46st-a) in *Hypostomus strigaticeps*, 2n=72 (10m+18sm+44st-a) in *Hypostomus regani* and 2n=76 (6m+16sm+54st-a) in *Hypostomus paulinus*. Ag-staining and FISH revealed various numbers and locations of NORs in the group. NORs were usually located terminally on the subtelocentric/acrocentric chromosomes: on the long arm in *Hypostomus strigaticeps* (2 to 4) and *Hypostomus paulinus* (2); and on the short arm in *Hypostomus ancistroides* (2 to 8) and *Hypostomus regani* (2 to 4). Conspicuous differences in heterochromatin distribution and composition were found among the species, terminally located in some st-a chromosomes in *Hypostomus ancistroides*, *Hypostomus strigaticeps*, and *Hypostomus paulinus*, and interstitially dispersed in most st-a chromosomes, in *Hypostomus regani*. The fluorochrome staining indicated that different classes of GC and/or AT-rich repetitive DNA evolved in this group. Our results indicate that chromosomal rearrangements and heterochromatin base-pair composition were significant events during the course of differentiation of this group. These features emerge as an excellent cytotaxonomic marker, providing a better understanding of the evolutionary mechanisms underlying the chromosomal diversity in *Hypostomus* species.

## Introduction

The suckermouth armored catfishes *Hypostomus* Lacépède, 1803 (Siluriformes, Loricariidae) represent one of the most specious genus of the family Loricariidae, with 127 nominal species (Zawadzki et al. 2008).

Most species of this family have a wide distribution in Central and South America. They usually dwell in the rapids, but may be present in different aquatic habitats and in sand banks or rocky rivers. The species of Hypostominae are restricted to freshwater habitats, with the exception of *Hypostomus* *watwata* Hancock, 1828,which is a benthic speciesthat lives in estuarine waters. Most of these animals have twilight habits and during daylight hours remain under stones or trunks of dead trees (Weber 2003).

The taxonomy of the Loricariidae family has constantly been reviewed through morphological studies (Reis et al. 2006), molecular phylogenies (Montoya-Burgos et al. 1998), allozymes (Zawadzki et al. 2005), and cytogenetic studies (Artoni and Bertollo 2001, Alves et al. 2006). In the most recent taxonomic study (Reis et al. 2006), this family was subdivided into six subfamilies: Lithogeneinae, Neoplecostominae, Hypoptopomatinae, Loricariinae, Hypostominae, and a new subfamily, Delturinae.

Among Hypostominae, only eight of its 30 genera (Armbruster 2004), namely *Ancistrus* Kner, 1854, *Hemiancistrus* Bleeker, 1862, *Hypostomus*, *Baryancistrus* Rapp Py-Daniel, 1989, *Panaque* Eigenmann and Eigenmann, 1889, *Pogonopoma* Regan, 1904, *Pterygoplichthys* Gill, 1858, and *Rhinelepis* Agassiz, 1829, have been object of cytogenetic studies. However, most of these reports are limited to the diploid number, silver staining of the nucleolus organizer regions (Ag-NORs), and chromosome C-banding (Artoni and Bertollo 2001, Alves et al. 2006). Among these genera, *Hypostomus* has the largest number of karyotyped species; however, the number of the studied species versus the species ascribed to the genus is scarce, i.e. approximately 10% (Table 1).

Concerning the cytotaxonomy, this genus shows a wide variation in diploid number, ranging from 2n=52 in *Hypostomus emarginatus* Valenciennes, 1840 (Artoni and Bertollo 2001) to 2n=84 in *Hypostomus* sp. 2-Rio Perdido NUP 4249 (Cereali et al. 2008). The most frequent diploid number was 2n=72 (Table 1). The occurrence of multiple NORs located in terminal position on the chromosomes is most common in this genus (Artoni and Bertollo 2001). Regarding the repetitive DNA in *Hypostomus*, different classes of GC and/or AT-rich heterochromatin, usually with segments located in terminal and/or interstitial chromosome regions, were observed in this fish group (Artoni and Bertollo 1999, Kavalco et al. 2004, Cereali et al. 2008).

The aim of this work was to analyze specimens of four species of the genus *Hypostomus* from different populations of the Paranapanema River Basin by means of conventional and molecular cytogenetic techniques and compare the obtained data with the cytogenetic records available for other species of the genus.

**Table 1.** A summary of cytogenetic data available for the genus *Hypostomus*.

**Species**	**Locality**	**2n**	**FN**	**KF**	**NORs**	**CB**	**Ref.**
*Hypostomus affinis* (Steindachner, 1877)	Jacuí stream (SP)	66	94	14m 14sm 12st 26a	5,t, la	t, la,pc	9,10
*Hypostomus albopunctatus* (Regan, 1908)	Mogi-Guaçu river (SP)	74	104	10m 20sm 44st-a	6,t,sa,la	n.d.	3
*Hypostomus albopunctatus*	Piracicaba river (SP)	74	104	10m 20sm 44st-a	3,t,sa,la	i,la,t,sa,pc	7
*Hypostomus ancistroides*	n.d.	68	106	10m 28sm 30st-a	n.d.	n.d.	2
*Hypostomus ancistroides*	Mogi-Guaçu river (SP)	68	102	16m 18sm 34st-a	6,t,sa	n.d.	3
*Hypostomus ancistroides*	Araquá river (SP)	68	96	18m 10sm 12st 28a	6,t,sa	n.d.	12
*Hypostomus ancistroides*	***	68	104	10m 26sm 32st-a	6,t,sa	t,la,pc	16
*Hypostomus* prope *auroguttatus* Kner, 1854	Mogi-Guaçu river (SP)	76	114	8m 30sm 38st-a	2,t,la	n.d.	3
*Hypostomus cochliodon* Kner, 1854	Salobra river and Salobrinha stream (MS)	64♂	100	16m 20sm 28st-a	n.d.	t,la	11
		64♀	97	16m 19sm 27st-a	n.d.	t,la	11
*Hypostomus emarginatus*	Araguaia river (MT)	52	98	16m 30sm 6st	2,t,la	n.d.	5
*Hypostomus goyazensis* (Regan, 1908)	Vermelho river (GO)	72	98	10m 16sm 10st 36a	2,t,sa	n.d.	12
*Hypostomus macrops* (Eigenmann et Eigenmann, 1888)	n.d.	68	92	10m 14sm 44st-a	n.d.	n.d.	2
*Hypostomus nigromaculatus* (Schubart, 1964)	Mogi-Guaçu river (SP)	76	104	8m 20sm 48st-a	3,t,la	t,la,pc	15
*Hypostomus nigromaculatus*	Três Bocas stream (PR)	76	102	6m 20sm 50st-a	3,t,sa,la	t,la,sa,pc	15
*Hypostomus paulinus*	n.d.	74	104	10m 20sm 44st-a	n.d.	n.d.	2
*Hypostomus paulinus*	Três Bocas and Apertados streams (PR)	76	98	6m 16sm 54st-a	2,t,la	t,la,pc	16
*Hypostomus plecostomus* (Linnaeus, 1758)		54	90	24m 12sm 18st-a	n.d.	n.d.	1
*Hypostomus regani*	Mogi-Guaçu river (SP)	72	102	10m 20sm 42st-a	n.d.	n.d.	3
*Hypostomus regani*	Araquá river (SP)	72	102	12m 18sm 26st 16a	4,t,la	n.d.	12
*Hypostomus regani*	Piumhi river (MG)	72	116	8m 16sm 48st-a	4,t,la	i	13
*Hypostomus regani*	Jacutinga river	72	100	10m 18sm 44st-a	4,t,sa	i,pc	16
*Hypostomus strigaticeps*	n.d.	74	86	8m 4sm 62st-a	n.d.	n.d.	2
*Hypostomus strigaticeps*	***	72	98	10m 16sm 46st-a	4,t,la	t,la,pc	16
*Hypostomus* sp. A	Córrego Rincão (SP)	70	102	18m 14sm 38st-a	4,t,sa,la	n.d.	3
*Hypostomus* sp. B	Mogi-Guaçu river (SP)	72	102	12m 18sm 42st-a	2,t,la	t,la,pc	3,4
*Hypostomus* sp. C	Mogi-Guaçu river (SP)	72	102	10m 18sm 44st-a	4,t,la	n.d.	3
*Hypostomus* sp. D1	Mogi-Guaçu river (SP)	72	108	10m 26sm 36st-a	4,t,la	n.d.	3
*Hypostomus* sp. D2	Mogi-Guaçu river (SP)	72	106	14m 20sm 38st-a	4,t,la	n.d.	3
*Hypostomus* sp. E	Mogi-Guaçu river (SP)	80	104	8m 16sm 56st-a	2,t,sa	t,la,sa,i,pc	3,4
*Hypostomus* sp. F	São Francisco river (MG)	76	102	10m 16sm 50st-a	n.d.	pc,t,i	4
*Hypostomus* sp. G	Araguaia river (MT)	64	102♂	14m 24sm 26st-a	2,sa	pc,t,i	6
		64	103♀	15m 24sm 25st-a	2,sa	pc,t,i	6
*Hypostomus* sp. 1	Paranapanema river (SP)	64	n.d.	n.d.	n.d.	n.d.	8
*Hypostomus* sp. 2	Alambari and Jacutinga streams (SP)	68	n.d.	n.d.	n.d.	n.d.	8
*Hypostomus* sp. 3	Quinta and Edgardia stream, Paranapanema river (SP)	72	n.d.	n.d.	n.d.	n.d.	8
*Hypostomus* sp. 4	Paranapanema river; Hortelã stream (SP)	76	n.d.	n.d.	n.d.	n.d.	8
*Hypostomus* sp. 2-rio Perdido NUP 4249	Perdido river (MS)	84	106	6m 16sm 62st-a	2,t,la	pc,t,la	14
*Hypostomus* sp. 3-córrego Salobrinha NUP 4247	Salobra river and Salobrinha stream (MS)	82	102	6m 14sm 62st-a	2,t,la	pc,t,la	14
*Hypostomus* sp. 1a	Patos stream (MG)	76	106	6m 8sm 62st-a	3,t,sa,la	t,la	13
*Hypostomus* sp. 1b	Araras stream (MG)	76	106	6m 8sm 62st-a	3,sa,la	t,la	13
*Hypostomus* sp. 2	Araras stream (MG)	74	106	10m 6sm 58st-a	2,la	t,la	13

Diploid numbers (2n), number fundamental (NF), karyotype formula (KF), metacentric (m), submetacentric (sm), subtelocentric (st) and acrocentric (a); *** several collection sites of the Paranapanema river basin. Number of nucleolar organizing region (NORs), C-banding (CB). Interstitial (i), terminal (t), pericentromeric (pc), short arm (sa), long arm (la). No data (n.d.). References (Ref.): (1) Muramoto et al. (1968), (2) Michele et al. (1977), (3) Artoni and Bertollo (1996), (4) Artoni and Bertollo (1999), (5) Artoni and Bertollo (2001), (6) Artoni et al. (1998), (7) Camilo (2004), (8) Fenerich et al. (2004), (9) Kavalco et al. (2004), (10) Kavalco et al. (2005), (11) Cereali (2006), (12) Alves et al. (2006), (13) Mendes Neto (2008), (14) Cereali et al. (2008), (15) Rubert et al. (2008), (16) Present study.

## Material and methods

Cytogenetic analysis was performed on a total of 148 specimens of four *Hypostomus* species collected at different sites of the Paranapanema River Basin (southern Brazil) (Table 1). The specimens were deposited in the Museu de Zoologia of the Universidade Estadual de Londrina (MZUEL), Londrina, Paraná State, Brazil.

### Conventional staining.

Metaphase chromosomes were obtained through the air-drying technique (Bertollo et al. 1978) and stained with 5% Giemsa stain solution (diluted with phosphate buffer, pH 6.8). The karyotypes were organized in groups of metacentric (m), submetacentric (sm), and subtelocentric-acrocentric (st-a) chromosomes.

### Chromosome banding.

C-banding was performed according to Sumner (1972). The silver staining of the nucleolus organizer regions (Ag-NORs) was performed according to Howell and Black (1980). The GC- and AT-rich bands were detected by staining with Chromomycin A_3_ (CMA_3_) and 4’6-diamidin-2-phenylindole (DAPI), respectively, according to Schweizer (1980). The slides were stained with 0.5 mg/mL CMA_3 _for 1 h, washed in distilled water and sequentially stained with 2 µg/mL DAPI for 15 min. Slides were mounted with a medium composed of glycerol/McIlvaine buffer (pH 7.0) 1:1 supplemented with 2.5 mM MgCl_2_.

### Fluorescence in situ hybridization (FISH).

The fluorescence in situ hybridization procedure was performed according to Swarça et al. (2001). The 18S rDNA probe of *Prochilodus argenteus* Spix and Agassiz, 1829 (Hatanaka and Galetti Jr 2004) was labeled with biotin-14-dATP by nick translation. Slides were treated with 30 µL of the hybridization mixture containing 100 ng of labeled probe (4 µL), 50% formamide (15 µL), 50% polyethylene glycol (6 µL), 20xSSC (3 µL), 100 ng of calf thymus DNA (1 µL) and 10% SDS (1 µL). The slides and the hybridization mixture were denatured at 90°C for 30 min in aTermocycler*,* and hybridization was performed overnight at 37°C in a humidified chamber. Post-hybridization washes were carried out in 2x SSC, 20% formamide in 0.1x SSC and 4xSSC/0.2% Tween 20, all at 42°C. The hybridized probe was detected with FITC-conjugated avidin. The post-detection washes were performed in 4xSSC/0.2% Tween 20 at RT. The slides were mounted in 23 µL DABCO solution consisting of the following: 90% glycerol, 2% Tris HCl 20 mM, pH 8.0, and 2.3% (wt/vol) 1,4-diazabicyclo (2,2,2) octane, pH 8.6), 1 μL of propidium iodate (1 μg/mL) and 1 µL of MgCl_2 _50 mM.

Images were acquired with Leica DM 4500 B microscope equipped with a DFC 300FX camera and Leica IM50 4.0 software.

## Results

**Figure 1.**
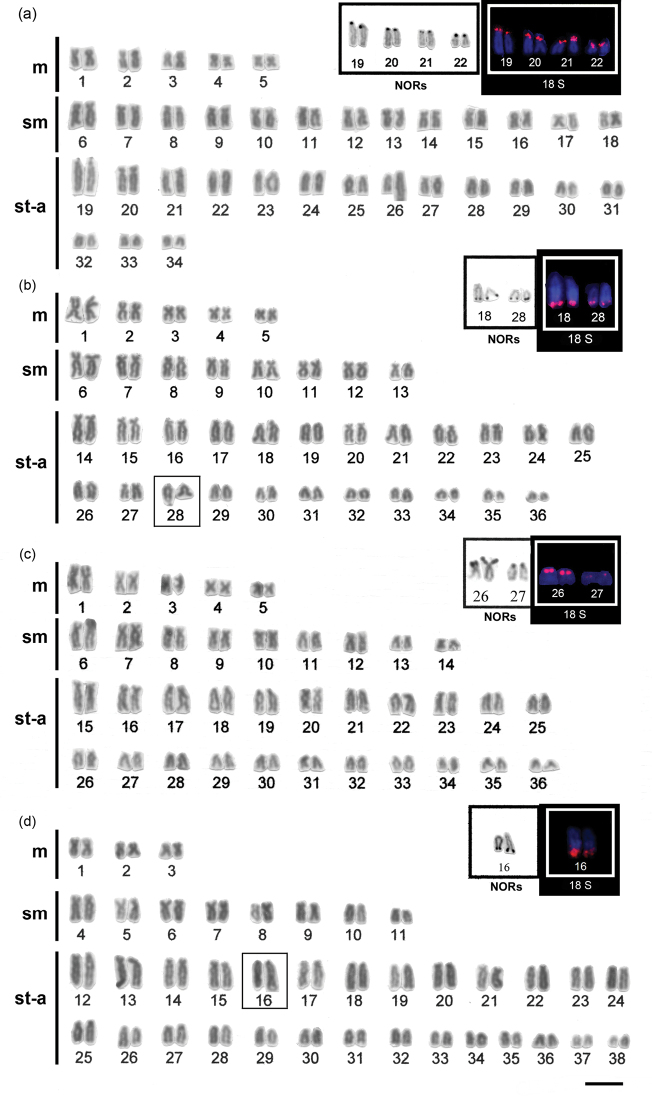
Karyotypes of **a** *Hypostomus ancistroides* **b** *Hypostomus strigaticeps* **c** *Hypostomus regani* **d** *Hypostomus paulinus* arranged from Giemsa-stained chromosomes. In the insets, partial karyotypes of the NOR-bearing chromosome pairs after Ag-staining (left) and FISH with 18S rDNA probe (right). Bar = 10 µm.

**Figure 2.**
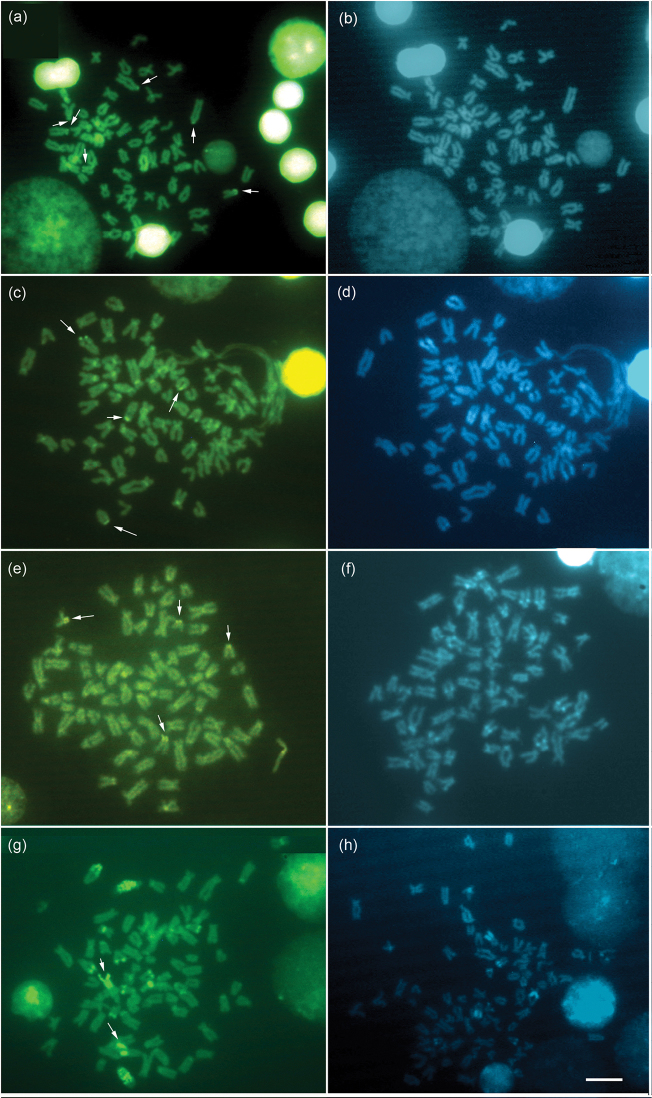
Metaphases stained with CMA_3_ (left) and DAPI (right), of *Hypostomus ancistroides* **a, b** *Hypostomus strigaticeps* **c, d** *Hypostomus regani* **e, f** *Hypostomus paulinus* **g, h**. The arrows indicate the NOR-bearing chromosomes. Bar = 10 µm.

**Figure 3.**
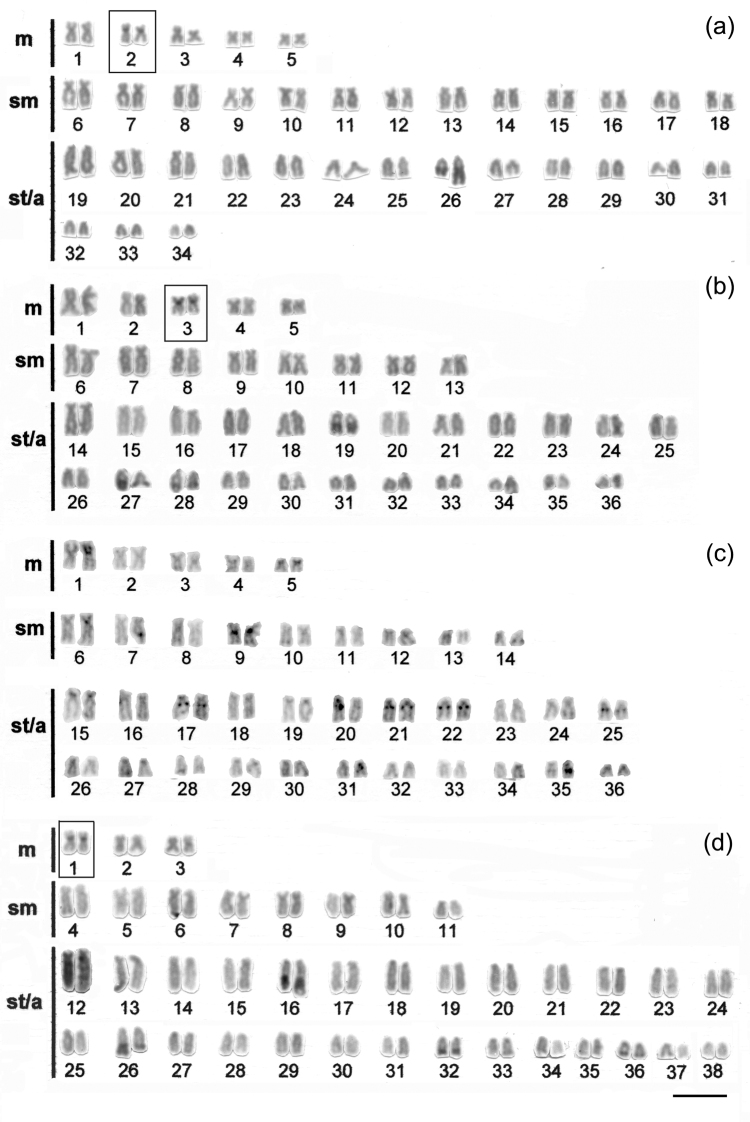
Karyotypes of **a** *Hypostomus ancistroides* **b** *Hypostomus strigaticeps* **c** *Hypostomus regani* and **d** *Hypostomus paulinus*, arranged from C- banded chromosomes Bar = 10 µm.

Specimens of *Hypostomus ancistroides* showed a diploid number 2n=68 and a fundamental number (FN) of 104, with a karyotype formula of 10m+26sm+32st-a. One chromosome of pair 26 showed size heteromorphism (Fig. 1a). Silver nitrate staining (Fig. 1a left box) and FISH (Fig. 1a right box) revealed up to four pairs of subtelocentric/acrocentric NOR-bearing chromosomes. CMA_3_ marked the terminal region of the long arms of pair 26, the pericentromeric region of the second pair of metacentric chromosomes, and probably the NOR-bearing chromosomes (Fig. 2a). No fluorescent staining was observed after DAPI staining (Fig. 2b). Heterochromatin was distributed in the pericentromeric region of the second pair (m) of the complement and in the terminal region of the long arm (pair 26) (Fig. 3a).

*Hypostomus strigaticeps* presented a diploid number 2n=72 and a FN of 98, with a karyotype formula of 10m+16sm+46st-a (Fig. 1b). The Ag-NOR site numbers ranged from two to four marked chromosomes (st-a) located in the terminal region of the long arm (pairs 18 and 28) (Fig. 1b left box), similar to the number observed in FISH (Fig. 1b right box). CMA_3_ marked four chromosomes, possibly the Ag-NOR sites, and the pericentromeric regions of most subtelocentric/acrocentric chromosomes (Fig. 2c). Staining with DAPI revealed large blocks in the terminal regions of four-eight subtelocentric/acrocentric chromosomes (Fig. 2d). C-banding revealed the occurrence of heterochromatic blocks in the pericentromeric region of the third pair of metacentric chromosomes and of up to eight large blocks in the terminal regions of the long arms of subtelocentric/acrocentric chromosomes. In one of those chromosome pairs, the heterochromatic block was adjacent to the secondary constriction (Fig. 3b).

*Hypostomus regani* had 2n=72 with a karyotype formula of 10m+18sm+44st-a and FN of 100 (Fig. 1c). Ag-NORs were located in the terminal position on the short arms of four subtelocentric/acrocentric chromosomes (pairs 26 and 27) (Fig. 1c left box). The same number of NOR-bearing chromosomes was observed after FISH (Fig. 1c right box) and CMA_3_-staining (Fig. 2e). Interstitial CMA_3_-negative blocks were observed in most of the subtelocentric/acrocentric chromosomes, which, in contrast, were positive after DAPI staining (Fig. 2f). Heterochromatin was distributed in the interstitial region of most st-a chromosomes and in the pericentromeric region of one metacentric pair (Fig. 3c).

*Hypostomus paulinus* showed 2n=76, FN=98 and a karyotype formula of 6m+16sm+54st-a (Fig. 1d). NORs were located in the terminal position on the long arms of chromosome pair 16 (Fig. 1d left box), similar to the chromosomes observed in FISH (Fig. 1d right box). CMA_3_-banding marked up to eight chromosomes (st-a) with large GC-rich blocks, and one st-a pair, probably corresponding to NOR-bearing chromosomes, and in the pericentromeric region of the first (m) pair (Fig. 2g); after DAPI staining, eight fluorescent bands were observed (Fig. 2h). Heterochromatin was distributed in the pericentromeric region of the first pair of metacentric chromosomes, in the terminal region of the long arms of eight pairs of subtelocentric/acrocentric chromosomes, one of which was the NOR-bearing pair. In this pair, a heterochromatin block was located at the proximal portion of the secondary constriction, whereas three heterochromatin blocks, which occupied almost the entire long arm, were observed in a pair of subtelocentric/acrocentric chromosomes (pair 12) (Fig. 3d).

## Discussion

All species differed with respect to their diploid chromosome number and/or karyotype, as follows: 2n=68 (10m+26sm+32st-a) in *Hypostomus ancistroides* (Fig. 1a), 2n=72 (10m+16sm+46st-a) in *Hypostomus strigaticeps* (Fig. 1b), 2n=72 (10m+18sm+44st-a) in *Hypostomus regani* (Fig. 1c), and 2n=76 (6m+16sm+54st-a) in *Hypostomus paulinus* (Fig. 1d). This variability is consistent with the chromosomal data previously reported in the genus *Hypostomus*, which showed a wide variation in 2n (from 52 to 84) (Table 1). The available cytogenetic studies showed that the species that possess the same 2n have different karyotypes. In the same way as the features observed in *Hypostomus ancistroides* (2n=68) but with different fundamental numbers (FN) among different populations, i.e. 106, 102 and 96 (Michele et al. 1977, Artoni and Bertollo 1996, Alves et al. 2006) and the characteristics found in *Hypostomus regani*, the cytogenetic analysis showed the same diploid number (2n=72) and a FN of 102 and 116 (Artoni and Bertollo 1996, Alves et al. 2006, Mendes Neto 2008), also differing from those analyzed herein (Table 1). On the other hand, studies conducted by Michele et al. (1977) in *Hypostomus paulinus* and *Hypostomus strigaticeps* showed differences in both 2n and FN. This difference may be ascribed to the existence of different cytotypes in these species, the occurrence of cryptic species, problems with the species identiﬁcation or with chromosomal classification.

According to Artoni and Bertollo (2001), 2n=54 is considered as a basal condition for the family Loricariidae. In a phylogenetic study of Loricariidae using morphological data, the genus *Hypostomus* was considered the most derived (Armbruster 2004), representing a group with more derived karyotypic forms, consisting mostly of st-a chromosomes with a high diploid number. It seems that there was a divergent karyotypic evolution among the *Hypostomus* species; on the other hand, two main chromosome rearrangements appear in the evolution of the genus: i) an increase in the diploid number (2n) in several species, probably due to centric fissions and ii) the same 2n but with a difference in the karyotype formula, probably accounted by pericentric inversions.

The same variability found in 2n and in karyotypes was also detected in NORs. Our data showed different phenotypes among the *Hypostomus* species, observed after silver staining and FISH. All species showed Ag-NORs and 18S rDNA sites located in the terminal regions of st-a chromosomes, but with a significant variation in number and location among them. *Hypostomus ancistroides* showed up to 8 NOR sites, all located on the short arms (Fig. 1a left and right boxes, respectively). *Hypostomus strigaticeps* showed NORs on the long arms and *Hypostomus regani*, NORs located on the short arms, and both species with up to 4 sites (Fig. 1b and 1c left and right boxes, respectively), and *Hypostomus paulinus* evidenced only two NOR-bearing chromosomes located on the long arm (Fig. 1d left and right boxes), which could be considered as species-specific characteristics.

The presence of one pair of NOR-bearing chromosomes, and also its interstitial location seems to be a widespread condition for Loricariidae fish, since this occurs among the Neoplecostominae and Hypoptopomatinae species (Alves 2000). However, in Hypostomini, the occurrence of multiple NORs and their location in the terminal position is most common, as observed here and recorded by other authors (Artoni and Bertollo 1996, Kavalco et al. 2005, Alves et al. 2006). But the exact location and number of ribosomal sites are confirmed only by the FISH technique. With regard to the genus *Hypostomus*, the available molecular cytogenetic data on the location of ribosomal genes are few and restricted to 18S rDNA sites of *Hypostomus affinis* (Kavalco et al. 2005). These data are very important to prompt more discussions about the evolution of ribosomal DNA in this group.

In the four species presently studied, the NORs were positive for CMA_3_ staining (Fig. 2), a feature that has been conserved among all Neoteleostei (Ráb et al. 1999). In addition, some other chromosomal regions were also considered GC-rich in the four species, mainly in *Hypostomus ancistroides* (Fig. 2a) and *Hypostomus paulinus* (Fig. 2g). *Hypostomus strigaticeps*, *Hypostomus regani*, and *Hypostomus paulinus* (Fig. 2d, f, h respectively) are three species that also showed several positive markers for DAPI staining, indicating AT-rich regions that were not found in *Hypostomus ancistroides* (Fig. 2b).

Some other studies carried out in *Hypostomus* (Artoni and Bertollo 1999, Kavalco et al. 2004, Cereali et al. 2008) also showed that this fish group may possess different classes of GC and/or AT-rich repetitive DNA families, as observed in the species analyzed in the present report. AT-rich regions are also rare among fishes, and have been reported mainly in some Hypostomini species (Artoni and Bertollo 1999, Kavalco et al. 2004, Rubert et al. 2008), some zebrafish species (Gornung et al. 1997, Phillips and Reed 2000), and gobiid fishes (Canapa et al. 2002).

The chromosome banding performed in all species analyzed showed a variation in the heterochromatin distribution pattern. However, the presence of heterochromatin in some chromosomes was constant, as observed in the pericentromeric region of a metacentric pair in *Hypostomus ancistroides* (pair 2), *Hypostomus strigaticeps* (pair 3), and *Hypostomus paulinus* (pair 1) (Fig. 3a, b, d, respectively), also reported in *Hypostomus nigromaculatus* by Rubert et al. (2008). An additional characteristic is the presence of some conspicuous blocks in the terminal regions of some st-a chromosomes of the karyotype. The same banding profile, organized in blocks, was also observed by others researchers: in *Hypostomus* sp. B from the Mogi Guaçu River (Artoni and Bertollo 1999), *Hypostomus affinis* (Kavalco et al. 2004), *Hypostomus cochliodon* (Cereali et al. 2008), and *Hypostomus nigromaculatus* (Rubert et al. 2008). Interestingly, in *Hypostomus paulinus*, pair 12 proved to be well differentiated, with the long arm almost entirely heterochromatic, a feature observed only in this species. On the other hand, *Hypostomus regani* showed a more distinct heterochromatin distribution in relation to the other species, with a preferential location in the interstitial regions of st-a chromosomes (Fig. 3c).

The presence of a marker chromosome that seems conserved for most *Hypostomus* species, corresponding to the NOR-bearing chromosome pair, which shows a heterochromatin block adjacent to this site (e.g. Artoni and Bertollo 1999, Kavalco et al. 2004, Rubert et al. 2008), was also observed. It can be inferred from all data on the heterochromatin composition and distribution that each species has its own peculiarities, i.e., each species has a unique banding pattern.

Karyotypes, banding patterns, number and location of ribosomal DNA sites, and repetitive DNA are important tools for the cytotaxonomy of *Hypostomus* species. Since these characteristics do not vary among the different populations of the same species, they are significant cytogenetic markers at the species level.

Further data on other *Hypostomu*s species from different rivers, as well as detailed studies of satellite DNA sequences may clarify important issues of genome organization, be used as genetic markers, and provide interesting insights for the comprehension of the evolution of this genus.
